# Application of Chemically Exfoliated Boron Nitride Nanosheets Doped with Co to Remove Organic Pollutants Rapidly from Textile Water

**DOI:** 10.1186/s11671-020-03315-y

**Published:** 2020-04-07

**Authors:** J. Hassan, M. Ikram, A. Ul-Hamid, M. Imran, M. Aqeel, S. Ali

**Affiliations:** 1 Solar Cell Applications Research Lab, Department of Physics, Government College University Lahore, Lahore, Punjab 54000 Pakistan; 2 Department of Physics, Riphah Institute of Computing and Applied Sciences (RICAS), Riphah International University, 14 Ali Road, Lahore, Pakistan; 3 Center for Engineering Research, Research Institute, King Fahd University of Petroleum & Minerals, Dhahran, 31261 Saudi Arabia; 4 State Key Laboratory of Chemical Resource Engineering, Beijing Advanced Innovation Centre for Soft Matter Science and Engineering, Beijing Engineering Center for Hierarchical Catalysts, Beijing University of Chemical Technology, Beijing, 100029 China

**Keywords:** Boron nitride, Exfoliation, Nanosheets, VSM

## Abstract

Two-dimensional layered materials doped with transition metals exhibit enhanced magnetization and improved catalytic stability during water treatment leading to potential environmental applications across several industrial sectors. In the present study, cobalt (Co)-doped boron nitride nanosheets (BN-NS) were explored for such an application. Chemical exfoliation process was used to exfoliate BN-NS and the hydrothermal route was adopted to incorporate Co dopant in various concentrations (e.g., 2.5, 5, 7.5, and 10 wt%). X-ray diffraction (XRD) study indicated that crystallinity improved upon doping with the formation of a hexagonal phase of the synthesized material. Selected area electron diffraction (SAED) confirmed enhanced crystallinity, which corroborates XRD results. Interlayer spacing was evaluated through a high-resolution transmission electron microscope (HR-TEM) equipped with Gatan digital micrograph software. Compositional and functional group analysis was undertaken with energy dispersive X-ray (EDS) and Fourier transform infrared (FTIR) spectroscopy, respectively. Field emission scanning electron microscope (FE-SEM) and HR-TEM were utilized to probe surface morphologies of prepared samples. Bonding modes in the sample were identified through Raman analysis. Optical properties were examined using UV-vis spectroscopy. Photoluminescence spectra were acquired to estimate the separation and recombination of excitons. Magnetic properties were studied by means of hysteresis loop acquired using VSM measurements. Methylene blue dye was degraded with as-prepared host and doped nanosheets used as catalysts and investigated through absorption spectra ranging from 250 to 800 nm. The experimental results of this study indicate that Co-doped BN-NS showed enhanced magnetic properties and can be used to degrade dyes present as an effluent in industrial wastewater.

## Introduction

Recently, boron nitride (BN), a promising two-dimensional layered material similar to graphene, tungsten disulfide (WS_2_), and molybdenum disulfide (MoS_2_), has attracted considerable attention. Boron nitride typically exists in its most stable crystallographic form, i.e., hexagonal boron nitride (h-BN). In addition, it is found as cubic boron nitride (c-BN) that is structurally analogous to diamond, rhombohedral boron nitride (r-BN), and an amorphous phase [1]. The interlayer spacing between h-BN layers is 3.30~3.34 Å while graphite exhibits a spacing of 3.33~3.35 Å. Besides, h-BN is a good insulator that possesses a bandgap of ~ 5.9 eV [2, 3]. The crystal structure of h-BN resembles that of graphene for which reason it is sometimes referred to as “white graphene” and is designated as graphene’s “twin material.” Interestingly, boron and nitrogen atoms are covalently bonded and arranged in a honeycomb-like pattern [2, 4]. Moreover, h-BN offers excellent physical, chemical, thermal, electrical, optical, and dielectric properties, which render it attractive for use in various applications [5–7]. Studies have been carried out to alter BN insulation characteristics through bandgap tuning and structural properties [3, 8]. Boron nitride nanosheets (BN-NS) were initially prepared in 2004 by exfoliating bulk material due to its unavailability in nature. To date, various methods have been adopted to produce nanosheets including chemical exfoliation [9], ball milling [10], electron beam irradiation [11], and chemical vapor technique [12]. Various other exfoliation routes are also described in the literature [13–15].

Potential applications of BN-NS include use in optoelectronics devices and thermal management devices. It is particularly suitable for use as a photocatalyst and catalyst in wastewater treatment [3, 16, 17]. Water plays a vital role in the survival and development of all living species on earth including humankind. An adequate supply of good quality water during all seasons has a major impact on the environment and economic growth of a region [18, 19]. Moreover, the food industry worldwide depends heavily upon a consistent supply of clean water [20]. The availability of pure and fresh water is influenced by many factors including the high rate of population growth. It is estimated that approximately 2.7 billion people in numerous countries face scarcity of clean water [18, 21].

According to literature, a large number of dyes including congo red, Martius yellow, methyl orange, methyl red, and methyl blue are used in a variety of industrial sectors such as leather, construction, paper, metal manufacturing, and printing [22–24]. Harmful metallic ions (Pb, Cr, Hg, Cu, etc.) produced due to the use of these dyes cause deleterious effects on human and aquatic life. Exposure to untreated dyes and toxic metallic ions can result in serious ailments such as anemia, cancer, encephalopathy, and weakening of the immune system [20, 25]. Furthermore, superfluous natural organic matter can increase the level of toxicity and adversely affect water purification systems [26].

Salt and other minor impurities can be removed from water by means of widely available techniques; however, the removal of harmful dyes and toxic metals ions is more challenging. A variety of routes have been utilized to purify water from these contaminants including photocatalysis [16], magnetic assistance [27], oil removal [28], and filtration and coagulation [29]. Among these techniques, catalysis holds an important place since it is considered environment-friendly, cost-effective, and energy-efficient. Furthermore, high surface area and superior chemical and physical properties of BN-NS make it suitable for use as a catalyst in wastewater treatment [17].

In the present study, magnetic properties of synthesized samples are also investigated due to its potential impact on the wastewater treatment process. Conventionally, transition metals that contain electrons in 3d or 4f shells are responsible for the origin of magnetism. Literature indicates that spontaneous magnetization is also observed in metal-free light elements which contain electrons in *s* and *p* orbital [30, 31]. Furthermore, the origin of ferromagnetism in diluted magnetic semiconductors or oxides (DMS(O)s) is heavily debated [32, 33]. Theoretical analysis suggests that periodic defects in graphene-based (2D) nanomaterials, particularly h-BN, induce magnetic ordering (ferromagnetism, ferrimagnetism, and anti-ferromagnetism) [34]. Moreover, these defects in h-BN act favorably to alter its diamagnetic behavior towards ferromagnetism [35]. Transition metals (e.g., Ni, Fe, Cu, Zn, and Co) exhibit satisfactory magnetic properties; therefore, doping of these species in BN nanosheets augurs promising results. Accordingly, doping of transition metal (Co) in h-BN gives rise to extrinsic defects which together with intrinsic defects serve to enhance its magnetic properties [36].

In the present study, a simple hydrothermal technique was used to prepare Co-doped BN nanosheets with enhanced catalytic activity and magnetic behavior. The effect of doping was investigated by evaluating the structural, morphological, optical, and magnetic properties of BN-NS.

## Methods

The current study was aimed to synthesize various concentrations of Co into h-BN nanosheets through the hydrothermal route to remove organic pollutants from textile water and magnetic behavior.

### Materials

Bulk BN powder (98%), dimethylformamide (DMF) methylene blue (MB), and sodium borohydride (NaBH_4_) were purchased from Sigma-Aldrich Co. (Germany). Cobalt (II) nitrate hexahydrate (CoH_12_N_2_O_12_) (98%) was purchased from VWR chemicals (UK). All chemicals acquired for this study were employed without additional purification.

### Exfoliation and Synthesis of Co-Doped BN

**Fig. 1:**
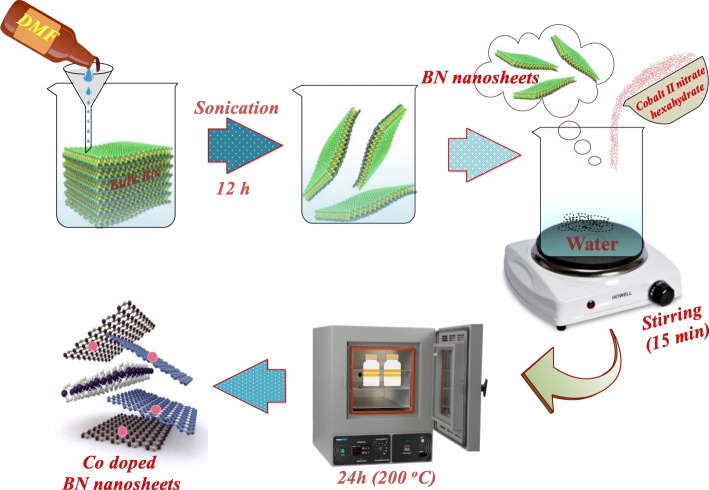
Schematic illustration of exfoliation and synthesis of Co-doped BN-NS

The chemical exfoliation route was employed to synthesize BN-NS. Firstly, 5 g bulk BN powder was dissolved in 200 mL DMF and stirred for 20 min to get the stock solution. This was followed by vigorous sonication for 12 h. After sonication, floated BN sheets were collected from the stock solution. Doping was undertaken via a hydrothermal approach. In this method, cobalt (II) nitrate hexahydrate was used as a Co dopant. Various cobalt (II) nitrate hexahydrate weight ratios (2.5, 5, 7.5, and 10 wt%) were doped on to the collected BN nanosheets. Afterwards, selected BN-NS and cobalt (II) nitrate hexahydrate with various ratios (1:0.25, 1:0.05, 1:0.075, 1:0.1) were dispersed in 100 mL deionized water under continuous stirring for 15 min. The suspension was poured into stainless steel autoclave, placed in a vacuum oven at 200 °C for 10 h as schematically presented in Fig. 1. Finally, autoclave was removed from the oven and cooled down at room temperature. The precursor was dried on a hot plate at 100–120 °C.

### Catalytic Activity

**Fig. 2:**
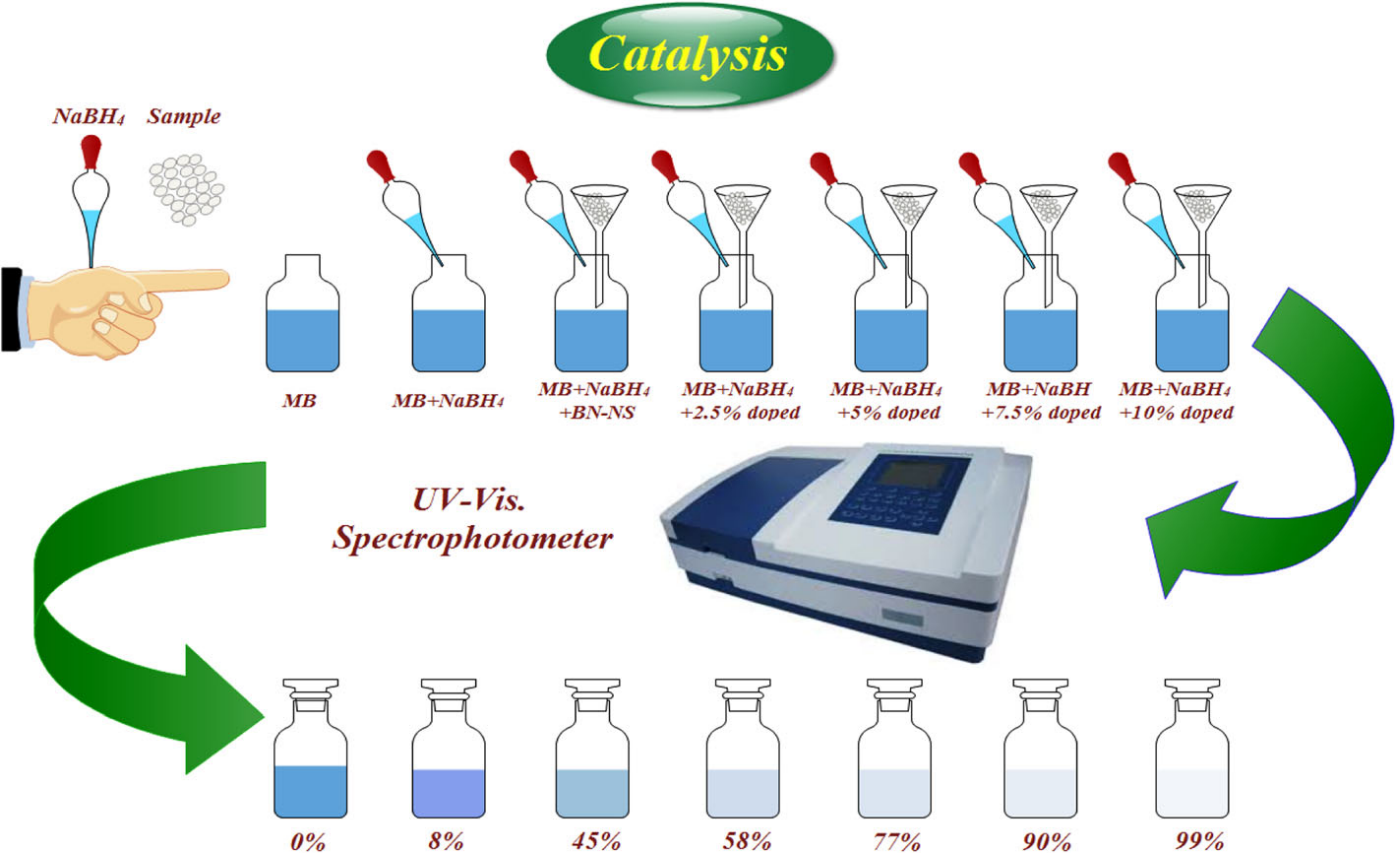
Schematic illustration of the experimental setup used to evaluate the catalytic activity

The catalytic activity of pure and Co-doped BN-NS was measured to determine the degree of dye degradation. This was undertaken by monitoring the degradation of MB in an aqueous solution of NaBH_4_ that serves as a reducing agent. Both MB and NaBH_4_ were freshly prepared to ensure the integrity of experimental data. Customarily, MB is the most commonly employed redox indicator in analytical chemistry to regulate catalytic activity during a dye degradation test. Additionally, MB remains blue in oxidized form whereas, it appears neutral when reduced [37]. Two catalytic experiments were performed, the first with 500 μL NaBH_4_ and 2 mg catalyst and the second with 1000 μL NaBH_4_ and 4 mg catalyst. In general, the concentration of a catalyst used in an experiment is the most significant factor that affects the chemical reaction. A catalyst lowers the activation energy (*E*_*a*_) of a reaction thereby serving to enhance its stability and rate of reaction. MB is primarily a toxic dye that is dangerous to the environment. It can be reduced by NaBH_4_ that converts it into a nontoxic and colorless species. However, the reduction process is relatively slow in the presence of NaBH_4_. Undoped and Co-doped BN-NS exhibit large surface area which when combined with an increase in reaction reactivity serves to accelerate the reduction efficiency of the dye. Incorporation of a catalyst into MB in the presence of a reducing agent causes adsorption. Additionally, a layer of reductant dispersed over catalysts may also accelerate adsorption due to the oxidation-reduction reaction between catalyst and MB. Reduction reaction by a catalyst occurs by transferring e^−^ from donor content BH_4_^−^ (e.g., from NaBH_4_) to acceptor content MB facilitated by pure and doped BN-NS. This results in the decrease of *E*_*a*_ which serves to stabilize the reaction. Catalytic activity was evaluated by taking 500 or 1000 μL of NaBH_4_ diluted in 10 mL of MB solution in a quartz cell. Afterwards, catalyst (2 or 4 mg) was added to investigate the degradation of MB. Dye degradation was evaluated spectrophotometrically as schematically illustrated in Fig. 2. Furthermore, absorption spectra ranging from 200 to 800 nm obtained with MB were used as a reference at room temperature. Degradation of dye in the presence of pure and doped catalyst confirmed enhanced catalytic activity, while NaBH_4_ failed to degrade MB.

### Material Characterizations

Prepared samples were analyzed using a variety of techniques. The phase constitution and degree of crystallinity were evaluated using PAN analytical X-pert PRO X-ray diffractometer (XRD) with Cu-Kα radiation (*λ* = 1.5418 Å) and 2θ ranging from 5° to 80°. Fourier transform infrared spectroscopy (FTIR) was performed using Perkin Elmer spectrometer to confirm the presence of functional groups. Emission spectra were obtained from photoluminescence (PL) spectroscope using JASCO FP-8200 spectrofluorometer. Raman spectra were obtained with DXR Raman microscope (Thermo scientific) having diode Laser at 532 nm. The morphological examination was conducted using JSM-6460LV field emission scanning electron microscope (FE-SEM) and Philips CM30 and JEOL JEM 2100F high-resolution transmission electron microscope (HR-TEM). Optical properties were recorded through UV-visible-Genesys 10S spectrophotometer. Energy-dispersive X-ray spectroscopy (EDS) was used to trace elemental composition. Magnetic properties were measured with a vibrating sample magnetometer (VSM).

## Results and Discussion

**Fig. 3:**
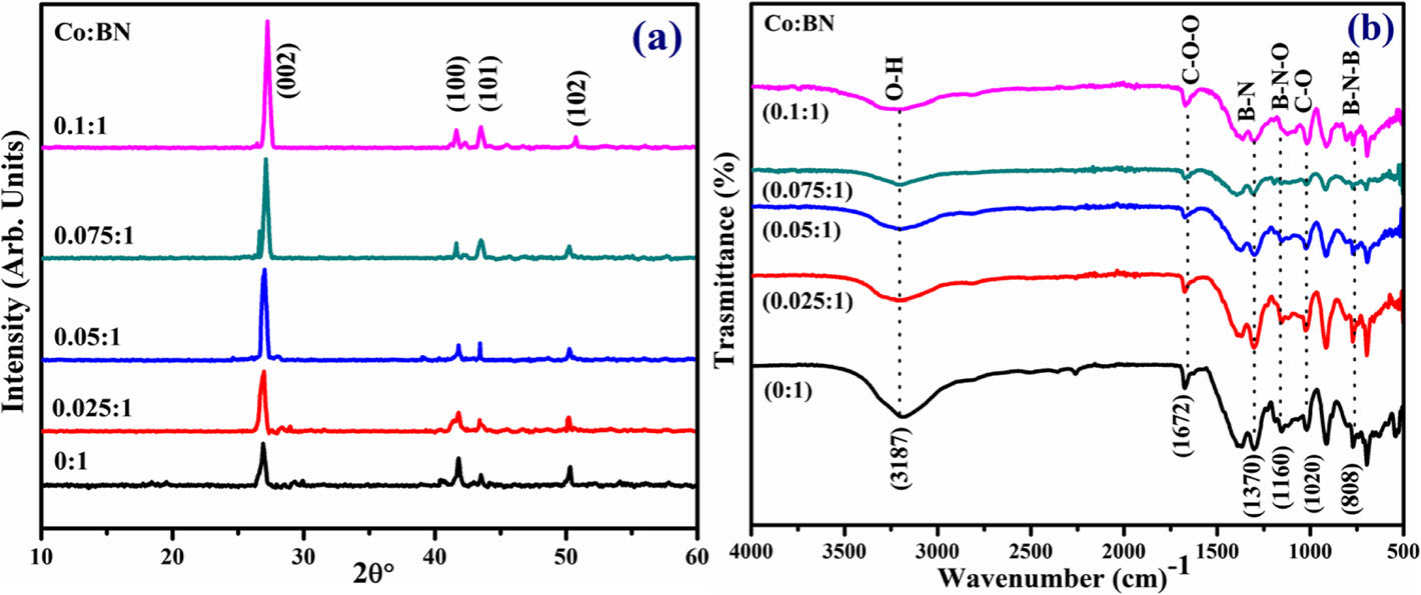
**a** XRD patterns of pristine and various concentrations (2.5, 5, 7.5, and 10 wt%) of Co-doped BN-NS. **b** FTIR spectra

XRD was used to analyze the phase and crystal structure of prepared samples, as illustrated in Fig. 3a. Diffraction peaks were observed at 26.8°, 41.6°, 43.52°, and 50.2° which were indexed as (002), (100), (101), and (102) planes, respectively. Observed reflections confirm the presence of a hexagonal phase of BN and agree well with JCPDS 00-034-0421 [38, 39]. It is worth noting that the characteristic peak intensity from pure to doped sample increases, which suggests that crystallinity was enhanced with the incorporation of Co. Additionally, XRD patterns indicate a peak shift towards a higher diffraction angle, which is attributed to the presence of dopant in the specimens [40]. Interlayer spacing *d*_002_ calculated with the help of Bragg’s law (nλ = 2dsinθ) was ~ 0.34 nm, which is consistent with HR-TEM results [41].

FTIR was performed to identify IR fingerprints in control and doped nanosheets, as illustrated in Fig. 3d. Spectra were observed at ~ 808, 1020, 1160, 1370, 1672, and 3187 cm^−1^. Two core peaks were identified at 808 and 1370 cm^−1^ which are thought to be associated with B–N–B (bending vibrations) and B–N (stretching vibration). The latter peak is associated with bending vibration A_2u_ mode (out-plane) while the former peak coincides well with stretching vibration E_1u_ mode (in-plane) [42, 43]. Furthermore, peaks at 1020, 1160, and 1672 cm^−1^ were consistent with C–O, B–N–O, and C=O bond, respectively [44]. Another peak centered at 3187 cm^−1^ corresponds to the B–OH bond [45, 46].

**Fig. 4:**
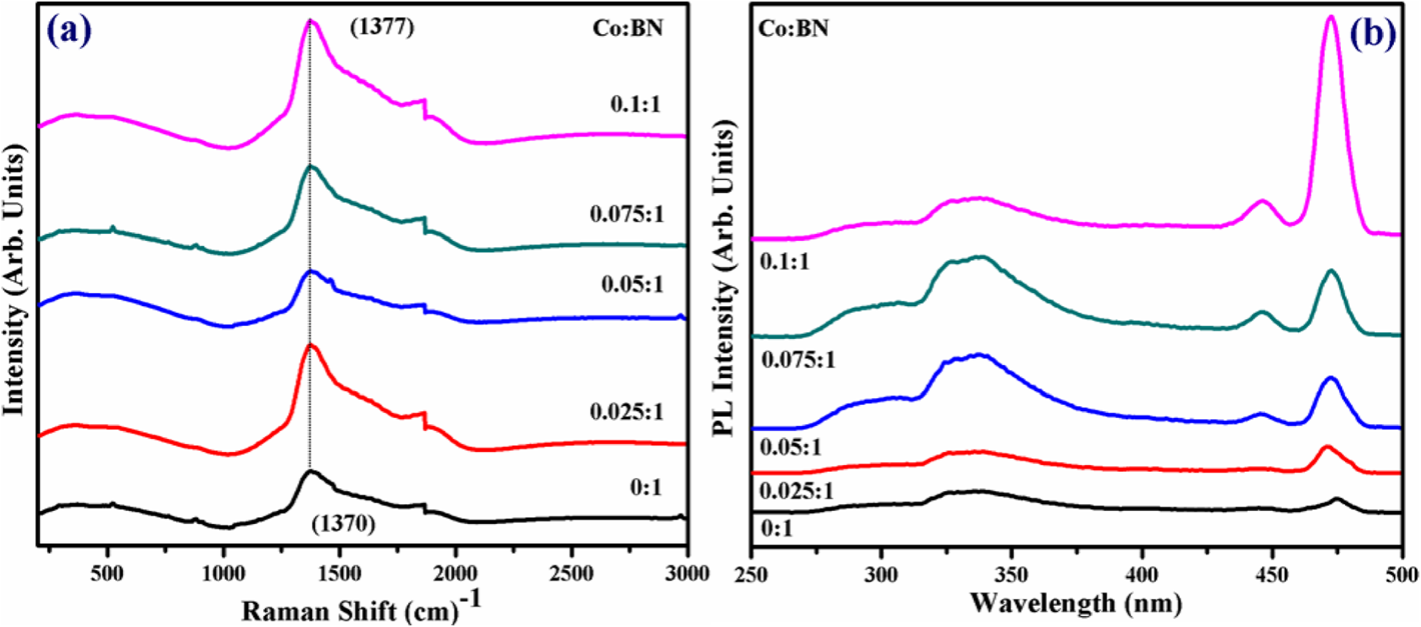
**a** Raman spectra of control and doped BN-NS. **b** PL spectra

The identification of structural fingerprints was carried out by Raman spectra, as illustrated in Fig. 4a. Spectra show characteristic Raman band centered at 1370 cm^−1^ which is ascribed to E_2g_ active phonon mode of h-BN and correlated to the G peak of graphene [47]. Exfoliated BN-NS exhibit minor peaks at 550 and 880 cm^−1^ which is attributed to fluorescent background [48]. Moreover, it is reported in the literature that high-quality mono-crystal h-BN exhibits E_2g_ active phonon mode at 1367 cm^−1^ [40]. In this study, red-shifted E_2g_ active phonon mode occurs as a result of faint interaction between interlayers of BN. Furthermore, red-shifted Raman spectra show the presence of a few layered nanosheets, which causes slight elongation within boron and nitrogen bonds (B–N) [49, 50]. Consequently, this elongation in B–N bond is due to the softening of phonons and agrees well with previously cited results. Additionally, elemental doping, ordering of stacked layers, domain size, and porosity could lead to broadening and shifting of peaks [51].

PL spectroscopy was performed to understand the excitonic migration and recombination of electron-hole pairs, as demonstrated in Fig. 4b. Spectra were observed with excitation and emission wavelength of *λ*_ex_ = 220 nm and *λ*_em_ = 310 nm, respectively. The characteristic band observed at ~ 322–342 nm corresponds to the electron-hole transition due to impurity level [52, 53]. It is noteworthy that the excitonic band increases but do not show wavelength shift upon doping. Characteristic peaks at ~ 446 nm and ~ 471 nm indicate that PL intensity shows a sharp increase from pure to doped samples. Meanwhile, 10 wt% Co-doped BN-NS has maximum PL intensity among all samples indicating maximum electron-hole recombination. The intensity gradually decreases due to doping concentration indicating separation of photo-generated charges [54]. Emission spectra revealed an excitation-dependent PL behavior that is consistent with the previously reported results [55].

**Fig. 5:**
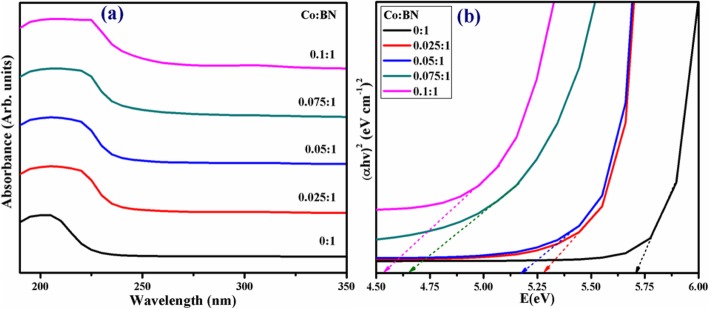
**a** UV-vis spectra of bare and Co-doped BN-NS. **b** Tauc-plot for bandgap

UV-vis spectroscopy was employed to investigate the absorption spectra and bandgap of as-prepared products, as shown in Fig. 5a. The characteristic absorption peak of the host BN-NS was at a threshold of ~ 205 nm in the deep ultraviolet region (DUV) referring to the bandgap of ~ 5.7 eV as presented in Fig. 5b. It is worth mentioning that bulk BN induces a bandgap of 5.2–5.4 eV while a monolayer exposes a bandgap of ~ 6.07 eV which coincides well with theoretical calculations (e.g., 6.0 eV). In the case of bi/multilayers, the bandgap value ranges from 5.56 to 5.92 eV [43]. Being consistent with a broad bandgap in Fig. 5b and DUV luminescence behavior in Fig. 5a, the h-BN nanosheets can be considered as a novel candidate for a variety of applications in photon emission, UV-lasing, and DUV detectors [56]. Furthermore, the absorption edge was shifted towards longer wavelengths with increasing doping concentrations (2.5 to 10 wt%) demonstrating a redshift (Fig. 5b) [57].

**Fig. 6:**
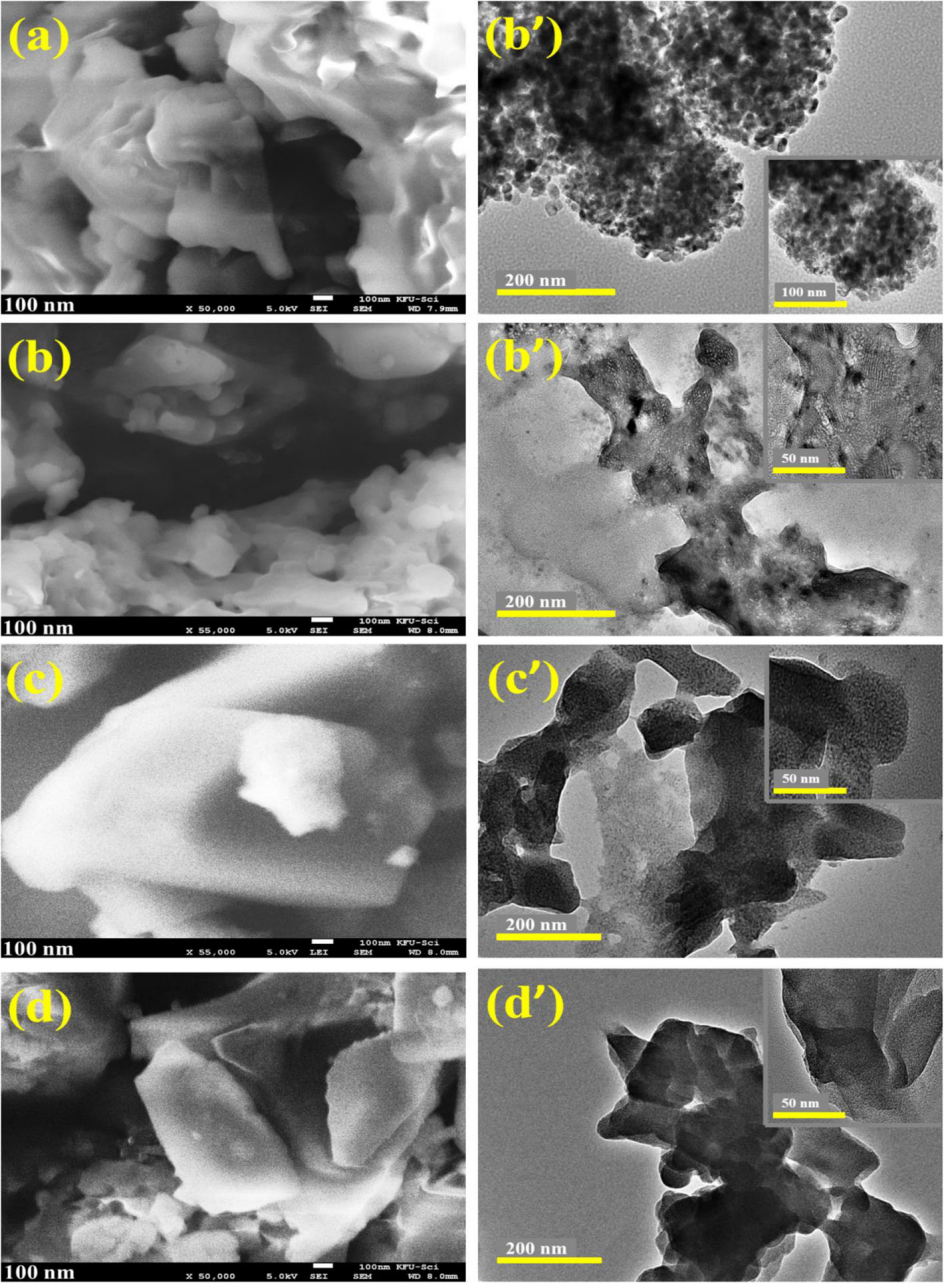
**a**, **a’** FE-SEM and HR-TEM images of pure BN-NS. **b**–**d** FE-SEM of (2.5, 7.5, and 10 wt%) doped BN-NS. **b’**–**d’** HR-TEM of (2.5, 7.5, and 10 wt%) doped BN-NS (inset 50 nm)

Morphology and composition of control and doped BN-NS were analyzed with FE-SEM as shown in Fig. 6a. The micrographs indicate that obtained particles possessed an aggregate nanosheet structure with a smooth surface and curved edges. Figure 6 b–d show BN nanosheets covered with cobalt. Agglomeration was observed in all samples. FE-SEM micrographs reveal that nanosheets are folded with a compact layered structure and possess a non-uniform shape and lateral dimension. Configuration and dimensions of nanosheets are a consequence of the exfoliation of bulk BN powder. The morphology of the obtained product was further confirmed with HR-TEM analysis. From HR-TEM micrographs shown in Fig. 6a–d, sheet-like morphology of as-prepared products was observed. Moreover, compact rough surfaces of nanosheets were observed with slightly porous features that results in enhanced catalytic activity. Dark spherical spots were observed indicating the successful incorporation of doping. Minute stacking and curled edges of nanosheets were recorded, as shown by SEM analysis in Fig. 6a–d. Experimental results suggest that FE-SEM and HR-TEM analysis point toward successful exfoliation of BN nanosheets from bulk BN.

**Fig. 7:**
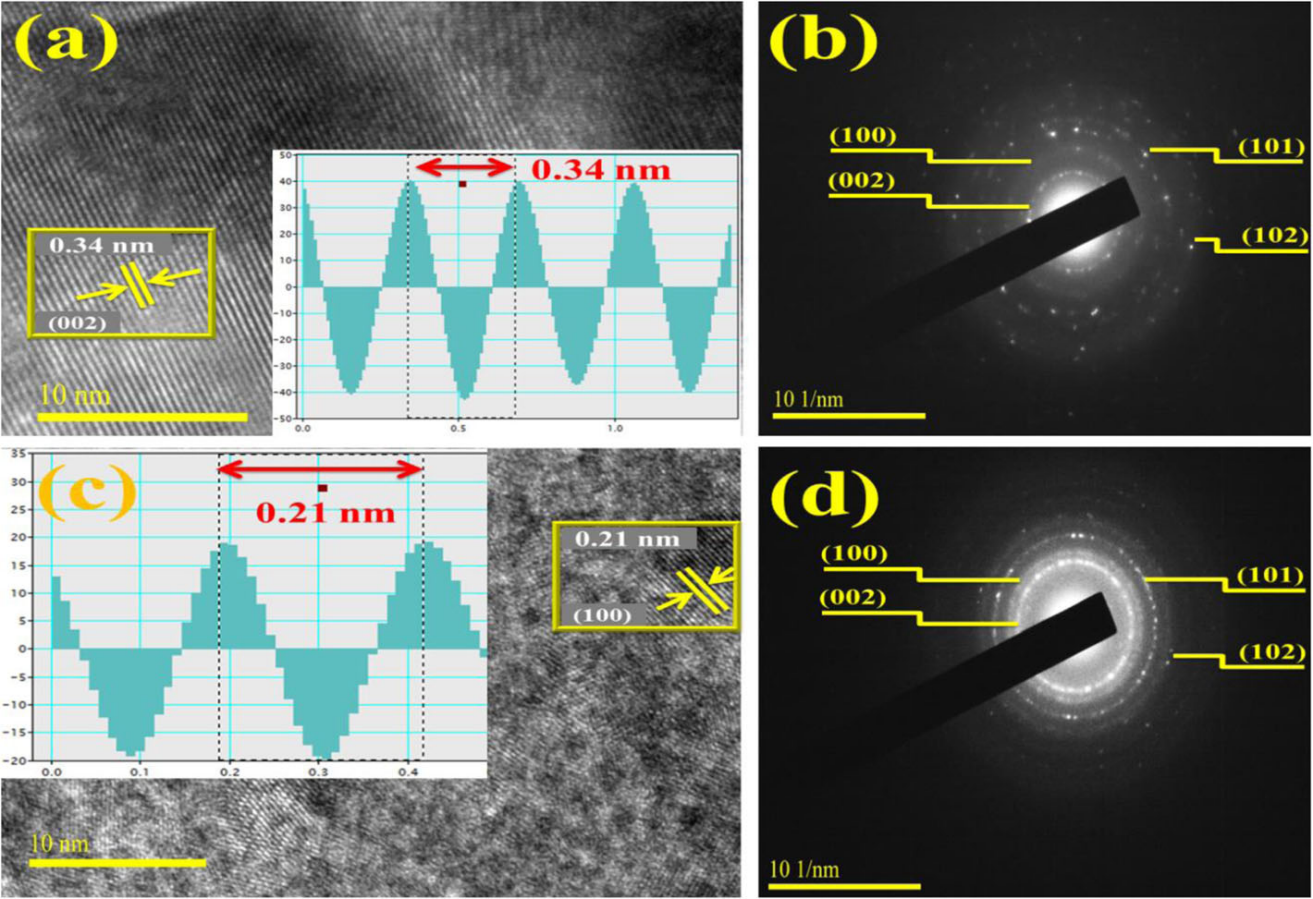
**a**, **c***d*-spacing analysis of host and 2.5 wt% doped BN-NS. **b**, **d** SAED patterns of control and 2.5 wt% doped BN-NS

Interlayer spacing measurements of bare and doped samples were undertaken with IFFT (see inset) induced by means of FFT images of HR-TEM examined through Gatan digital micrograph software to distinguish lattice fringes. Observed *d*-spacing value for pristine and 2.5 wt% doped BN-NS is 0.34 nm and 0.21 nm which corresponds to *d*_002_ and *d*_100_ planes, respectively, as illustrated in Fig. 7a, c. These findings agree well with XRD analysis and standard data [58]. Furthermore, SAED profiles are demonstrated in Fig. 7b, d which signify bright spot diffraction rings. These diffraction rings were indexed as originating from (002), (100), (101), and (102) planes that agree well with XRD results. The SAED patterns suggest that all rings belong to hexagonal BN and validate the highly crystalline nature of nanosheets [58].

**Fig. 8:**
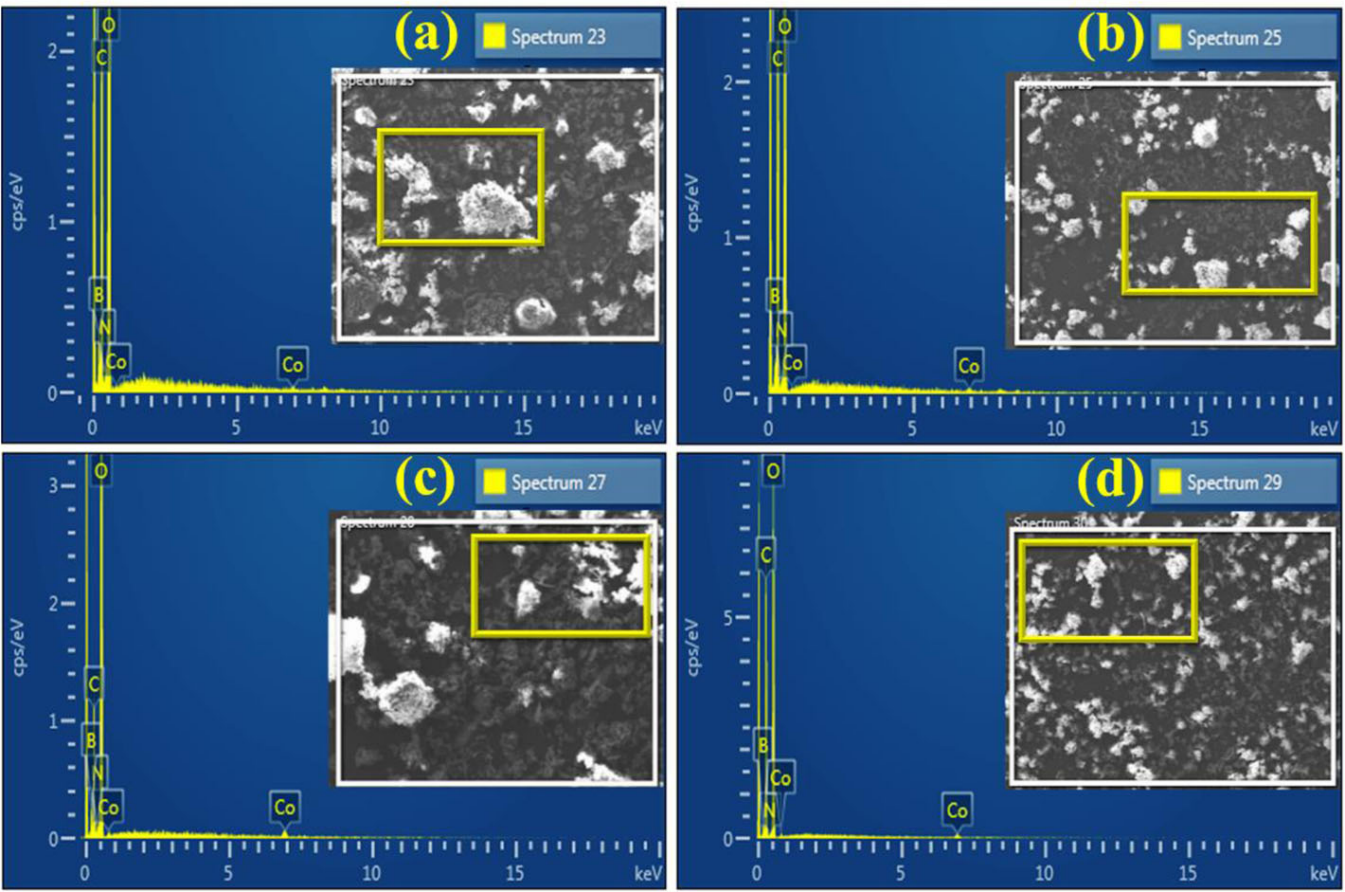
**a**–**d** EDS analysis of various concentrations (2.5, 5, 7.5, and 10 wt%) of Co-doped BN-NS

The surface elemental composition of doped BN-NS was investigated through EDS analysis as shown in Fig. 8a–d, respectively. Obtained micrographs exhibit strong peaks of boron (B) and nitrogen (N) whereas minor signal for cobalt (Co) was also observed in the EDS spectrum (Fig. 8a). Two moderate peaks of Co at 0.5 and 7 keV were observed in Co-doped samples, which confirm the successful incorporation of the dopant. In addition, the carbon signal below 1 keV originates from carbon tabs used to hold the sample during analysis and/or is due to high background counts in the SEM-EDS detector. Otherwise, there was no carbon in the sample [59].

**Fig. 9:**
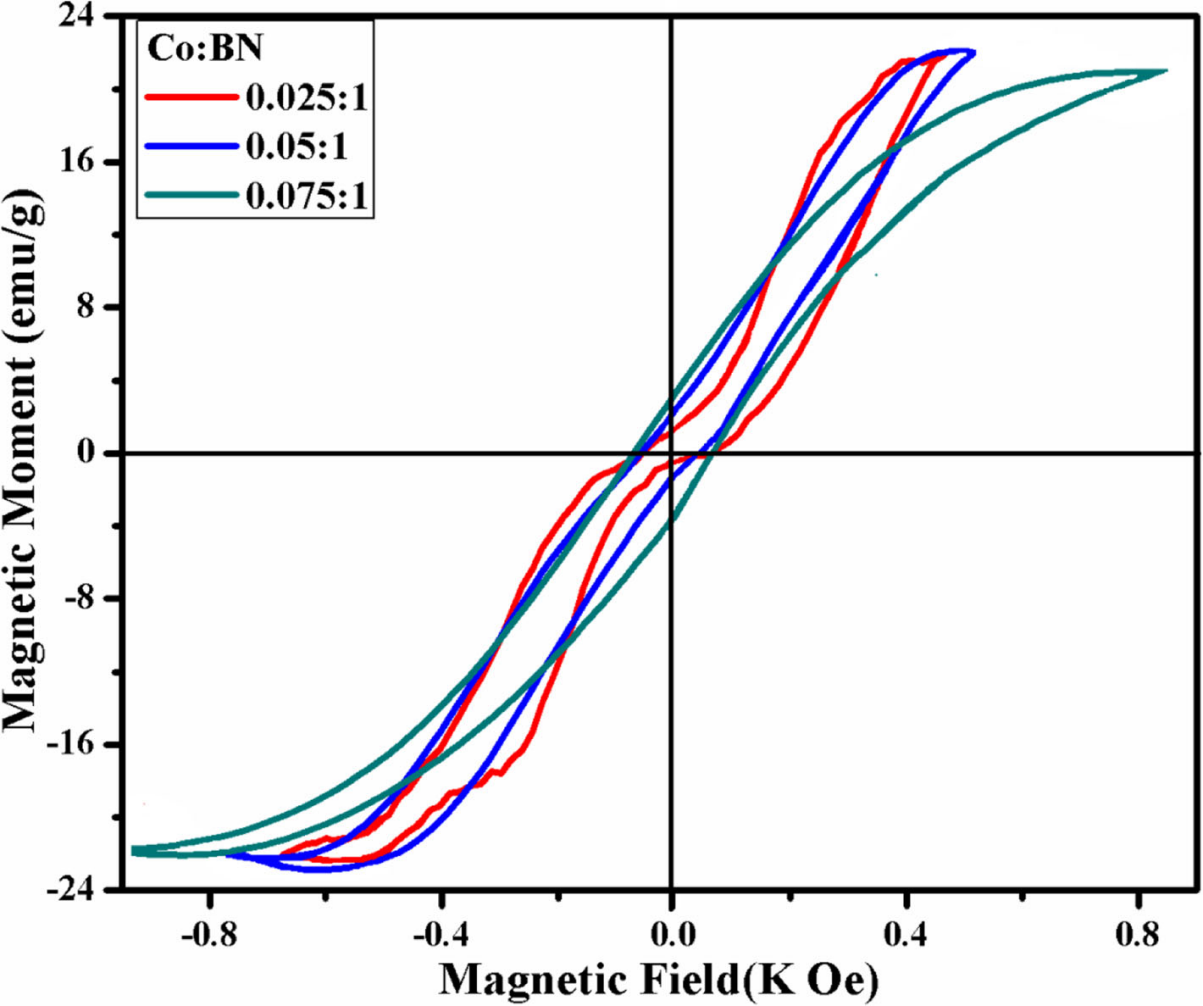
M–H curve of various concentration (2.5, 5, and 7.5 wt%) of Co-doped BN-NS

Magnetic properties of Co-doped BN-NS were evaluated with M–H curve using VSM measurements. In the graph shown in Fig. 9, the sigmoidal appearance of M–H loops demonstrates that Co-doped BN-NS is characterized by magnetic moment. Literature study reveals that pristine BN shows diamagnetic behavior with susceptibility (χ ≈ − 8.6 × 10^−7^ emu/g) [31, 35]. In contrast, Co-doped BN-NS exhibits room temperature ferromagnetism (RT-FM) resulting from exchange interaction among Co^+2^ ions and unpaired dipoles that tend to align along the applied magnetic field. It can be seen that the hysteresis loop appears more precise as well as doping concentration increases, which affirms purity and successful incorporation of the dopant. The coercivity, remanence, and saturation magnetization of Co-doped BN-NS predict softness and hardness of the magnetic material. Co-doping in BN-NS results in the formation of a soft magnetic material. The values of remanence (*M*_*r*_), saturation magnetization (*M*_*s*_), and coercivity (*H*_*C*_) for various doping concentrations (2.5, 5, and 7.5 wt%) were calculated by M–H curve as demonstrated in Table 1.

**Table 1:** Magnetic properties of various Co-doped BN-NS samples

Samples (doped BN-NS)	Coercivity (*H*_*c*_) (Oe)	Remanence magnetization (*M*_*r*_) (emu/g)	Saturation magnetization (*M*_*s*_) (emu/g)	Squareness ratio (*M*_*r*_/*M*_*s*_)
2.5%	54.8	1.23	22.04	0.055
5.0%	57.5	2.05	22.03	0.093
7.5%	69.4	2.95	20.89	0.141

**Fig. 10:**
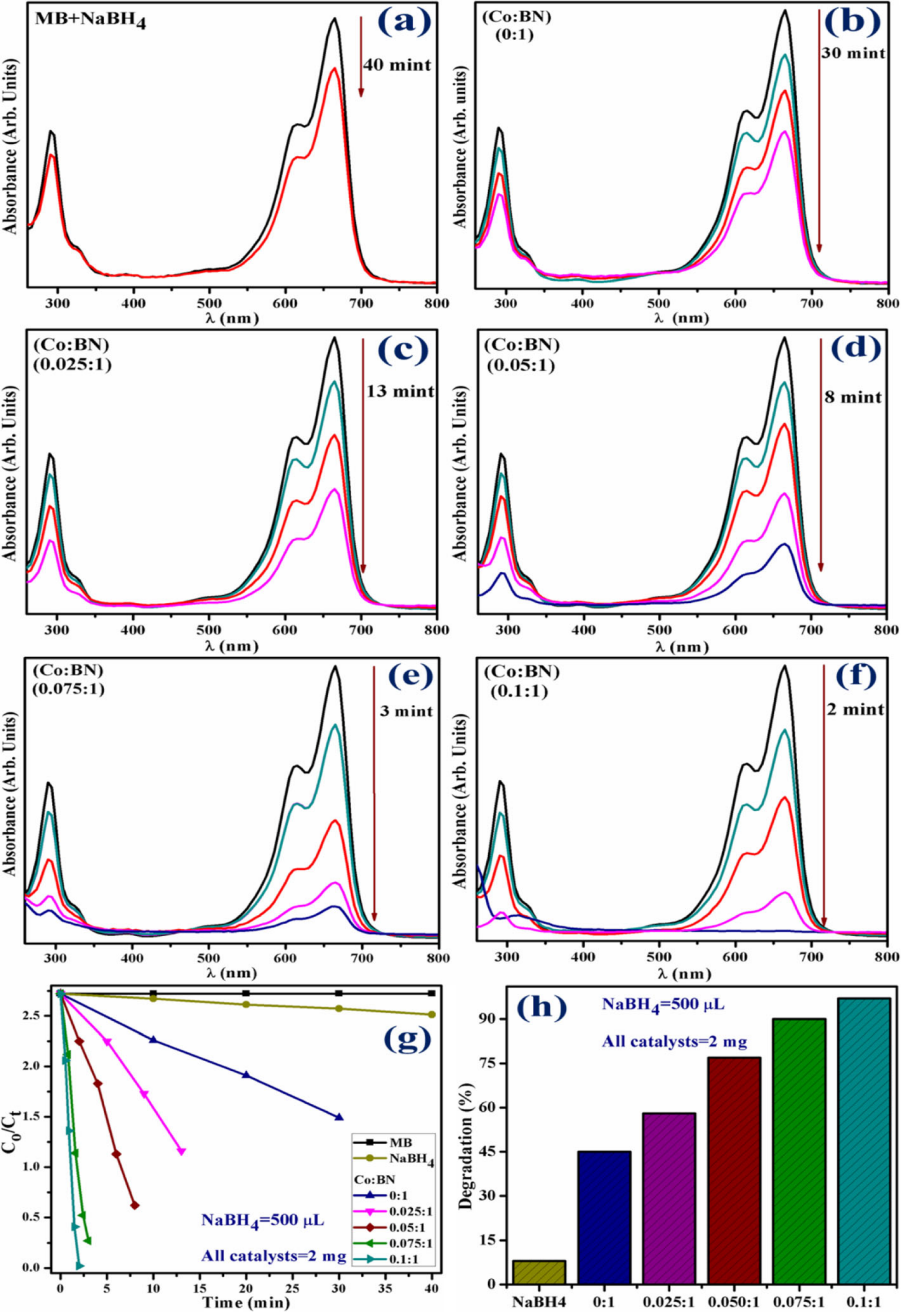
**a** Time-dependent UV-vis spectra of NaBH_4_. **b** pristine BN-NS. **c**–**f** Various concentrations (2.5, 5, 7.5, and 10 wt%) of Co-doped BN-NS. **g** Plots of *C*_*t*_/*C*_*o*_ versus time. **h** Comparison of degradation percentage over various concentrations of BN-NS

The catalytic activity of pristine and Co-doped BN-NS which works as catalysts was expressed by the degradation of MB and investigated through absorption spectra monitored with UV-vis spectrophotometer. Figure 10 a–h show the results of catalytic activity using 500 μL of NaBH_4_ and 2 mg catalyst. From Fig. 10a, it can be seen that NaBH_4_ fails to degrade MB successfully, as it degrades only 8% of the dye after 40 min. Incorporation of pure BN-NS into MB in the presence of NaBH_4_ resulted in 45% degradation in 30 min (Fig. 10b). Moreover, the degradation capability of Co-doped BN-NS (see Fig. 10b–e) was significantly higher. Various concentrations (2.5, 5, 7.5, and 10 wt%) of doped catalyst demonstrate 58, 77, 90, and 97% degradation in 13, 8, 3, and 2 min, respectively. Interestingly, 10 wt% doped nanosheets yield superior catalytic activity and degrade 97% dye in just 2 min which is higher than (7.5, 5, and 2.5 wt%) doped concentrations and characteristic peak intensity is observed at ~ 290 and 665 nm. This increment in the dye degradation is possibly due to the enhancement of available adsorption as well as catalytic sites on catalysts. In this mechanism, 3d state of Co interacts well with 2p state of corresponding available B or N sites in BN-NS. This strong interaction among 3d Co and 2p B or N states causes to enhance catalytic activity and result in rapid degradation of dye, which favorably supports our results [60].

**Fig. 11:**
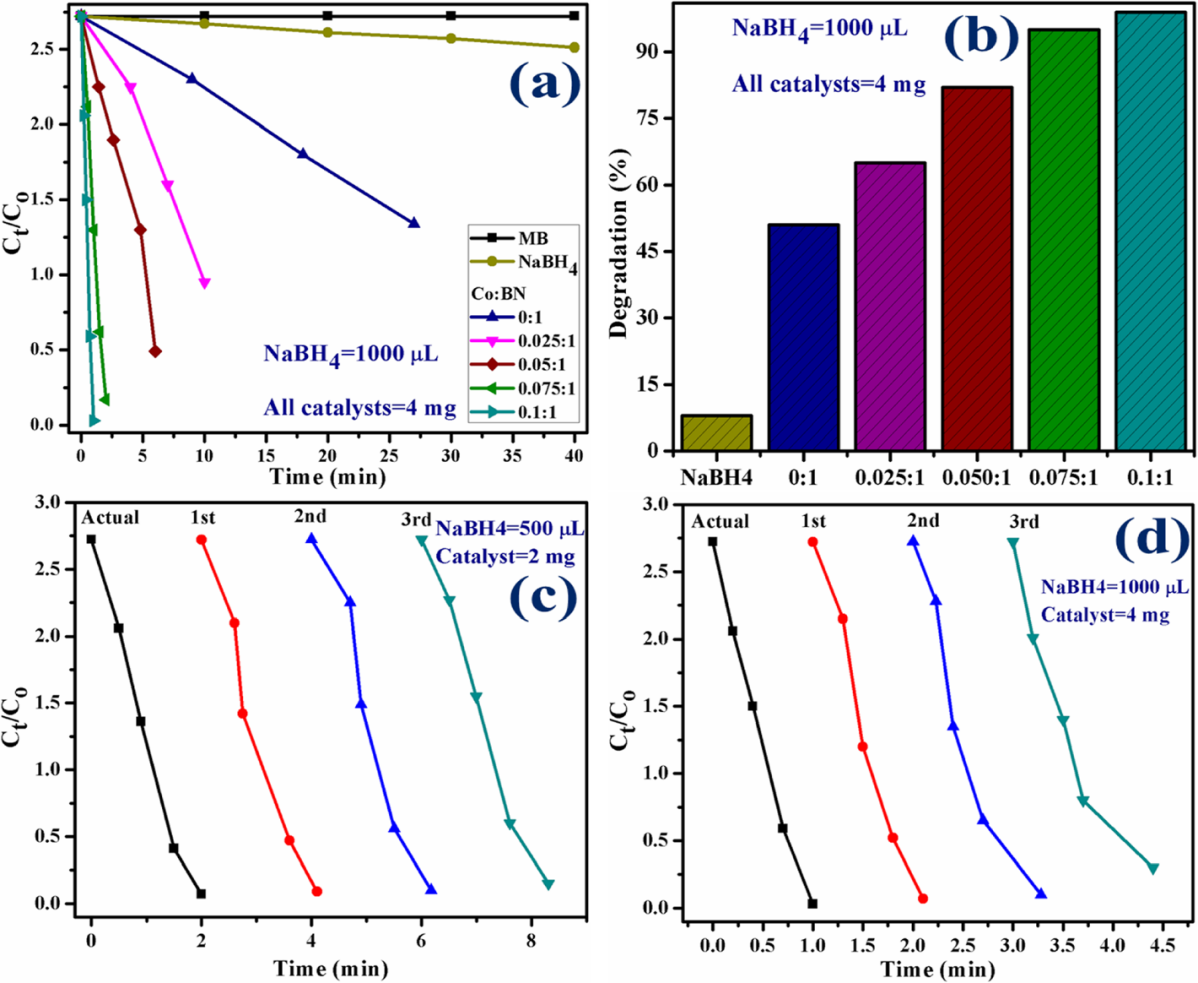
**a** Plots of *C*_*t*_/*C*_*o*_ versus time using NaBH_4_ = 1000 μL and catalysts = 4 mg. **b** Comparison of degradation percentage over various concentrations. **c** Plots of *C*_*t*_/*C*_*o*_ versus time for reusability of 10 wt% Co-doped BN-NS. **d** Plots of *C*_*t*_/*C*_*o*_ versus time for reusability of 10 wt% Co-doped BN-NS

The catalytic experiment was repeated using 1000 μL of NaBH_4_ and 4 mg of catalyst. By increasing the concentration of the catalyst, the reaction proceeds more quickly as compared to the previously mentioned experiment. This observation agrees well with the literature. In the present experiment, NaBH_4_ still failed to degrade MB meanwhile pristine BN-NS and various concentrations (2.5, 5, 7.5, and 10 wt%) of doped catalyst degraded 51, 65, 82, 95, and 99% in 27, 10, 6, 2, and 1 min, respectively, as evaluated with a spectrophotometer. Experimental results measuring catalytic activity are illustrated in Fig. 11a, b.

According to the Beer-Lambert law, the ratio of the concentration of MB at a specific time (*C*_*t*_) and initial concentration of MB (*C*_*o*_), referred to as *C*_*t*_/*C*_*o*_, can be estimated by the ratio of parallel absorbance (*A*_*t*_/*A*_*o*_). Figures 10 f and 11 a depict the time-course of *C*_*t*_/*C*_*o*_ used for all catalysts while Fig. 10 g and 11 b indicate the percentage degradation of all catalysts. The percentage of degradation was evaluated by Eq. 1.

1 $$ \mathrm{Degradation}\ \left(\%\right)=\frac{Co- Ct}{Co}\times 100 $$

Furthermore, the pH value is a vital operational variable in dye degradation treatment. Also, pH has an important role in textile wastewater treatment and in the reaction mechanisms that contribute to dye degradation. It is worth mentioning that percentage of degradation depends on the pH value to a significant extent. In the present experiment, the pH value was set at 8.5. Significantly, dye degradation due to catalytic activity demonstrated best results in an alkaline environment which favorably supports our experimental findings. Several reports reveal that the highest dye degradation by catalytic activity was observed in an alkaline environment [61].

Stability and reusability (recycling ability) of catalysts is an important characteristic to evaluate a catalyst used for dye degradation. In the present study, the stability of catalysts was evaluated by allowing the performed experiment to stay for 48 h. After 48 h, results were the same as performed initially, i.e., degradation was still in its previous condition. Reusability of catalyst was investigated by recycling 10 wt% Co-doped catalyst which served as a superior catalyst in the present study for three cycles. Extracted spectra of recycled catalytic activity were evaluated as shown in Fig. 11c, d.

In addition, the load of the catalyst before and after three times of recycling process was examined. In the first and second activity, minor weight loss of catalyst ranging from 2 mg and 4 mg (before) to 1.7 mg and 3.6 mg (after three cycles) was detected by considering ~ 5% sensing deviation in the present experiment. These findings indicate that Co-doped BN-NS exhibit outstanding stability while serving as a catalyst. Finally, this study suggests that Co-doped BN-NS exhibits an efficient and outstanding catalytic behavior toward dye degradation in industrial wastewater treatment.

## Conclusion

In this study, boron nitride nanosheets (BN-NS) were synthesized through chemical exfoliation of bulk BN powder. Various concentrations (2.5, 5, 7.5, and 10 wt%) of Co were successfully incorporated via the hydrothermal approach. As prepared, pure and doped BN-NS were characterized by a variety of techniques to evaluate the effect of doping. XRD patterns confirm the presence of the hexagonal phase of BN with improved crystallinity from pure to doped samples. Furthermore, the peak shift indicates the successful incorporation of doping. FTIR spectra indicate sp^2^ bonded B–N stretching vibrations consistent with E_1g_ mode (in-plane) as well as B–N–B bending vibration associated with A_2u_ mode (out plane). Raman spectroscopy affirmed E_2g_ active phonon mode of h-BN while photoluminescence spectroscopy revealed emission spectra that were attributed to exciton migration and recombination. Host and Co-doped BN-NS displayed absorbance in the DUV region along with a redshift that causes a decrease in bandgap energy suggesting it to be a suitable material for degradation of dye from industrial wastewater and organic pollutants. Sheet-like morphology of obtained product was studied by means of FE-SEM and HR-TEM. Slightly porous features result in high catalytic activity due to available adsorption sites. EDS analysis showed the purity of the sample and confirmed the incorporation of dopant in nanosheets. The magnetic behavior of Co-doped BN-NS was investigated through VSM measurements that display strong ferromagnetic behavior while pristine BN-NS show diamagnetic behavior. Significantly, the sigmoidal appearance of the hysteresis loop becomes more precise from lower to a higher concentration of Co-doped BN-NS, which point toward the formation of a soft magnetic material. Lastly, pure and Co-doped BN-NS was utilized as a catalyst in dye degradation. The catalytic activity provides efficient results for most samples but 10 wt% Co-doped catalyst showed significant outcome with the highest dye degradation (99%) in 1 min, making it a novel catalyst in this study. Extracted results from pure and doped BN-NS can be used as a guideline to modify and enhance magnetic properties in order to improve reliability in modern optoelectronic technology. Finally, the synthesized material has the potential to be used as a stable, reusable, and superior nano-catalyst to replace conventional wastewater treatment methods.

## Data Availability

All data are fully available without restriction.

## Abbreviations

BN-NS: Boron nitride nanosheets
Co: Cobalt
UV-vis: Ultra-violet visible spectroscopy
XRD: X-ray diffraction
DUV: Deep ultraviolet region
FTIR: Fourier transform infrared spectroscopy
PL: Photoluminescence
DMF: Dimethylformamide
MB: Methylene blue
NaBH_4_: Sodium borohydride
EDS: Energy-dispersive X-ray spectroscopy
FE-SEM: Field emission scanning electron microscopy
HR-TEM: High-resolution transmission electron microscopy
JCPDS: Joint committee on powder diffraction standards,
VSM: Vibrating sample magnetometer measurements
